# Incidence and predictors of hypoglycaemia in type 2 diabetes – an analysis of the prospective DiaRegis registry

**DOI:** 10.1186/1472-6823-12-23

**Published:** 2012-10-17

**Authors:** Diethelm Tschöpe, Peter Bramlage, Christiane Binz, Michael Krekler, Evelin Deeg, Anselm K Gitt

**Affiliations:** 1 Stiftung “Der herzkranke Diabetiker” in der Deutschen Diabetes Stiftung, Bad Oeynhausen, Germany; 2 Herz- und Diabeteszentrum Nordrhein-Westfalen, Universitätsklinik der Ruhr Universität Bochum, Bad Oeynhausen, Germany; 3 Institut für Pharmakologie und präventive Medizin, Mahlow, Germany; 4 Bristol-Myers Squibb, Medical Department, Munich, Germany; 5 Institut für Herzinfarktforschung Ludwigshafen an der Universität Heidelberg, Ludwigshafen, Germany; 6 Herzzentrum Ludwigshafen, Medizinische Klinik B, Kardiologie, Ludwigshafen, Germany

## Abstract

**Background:**

Hypoglycaemia is a serious adverse effect of antidiabetic drug therapy. We aimed to determine incidence rates of hypoglycaemia in type-2 diabetic patients and identify predictors of hypoglycaemia when treatment is intensified.

**Methods:**

DiaRegis is a prospective German registry that follows 3810 patients with type-2 diabetes referred for treatment intensification because of insufficient glycaemic control on one or two oral antidiabetic drugs.

**Results:**

Out of a total of 3347 patients with data available for the present analysis 473 (14.1%) presented any severity hypoglycaemia over a follow-up of 12 months. 0.4% were hospitalized (mean of 1.3±0.6 episodes), 0.1% needed medical assistance (1.0±0.0), 0.8% needed any help (1.1±0.5) and 10.1% no help (3.4±3.7), and 8.0% had no specific symptoms (3.6±3.5). Patients with incident hypoglycaemia had longer diabetes duration, higher HbA1c and a more frequent smoking history; more had co-morbid disease conditions such as coronary artery disease, peripheral arterial disease, amputation, heart failure, peripheral neuropathy, diabetic retinopathy and clinically relevant depression at baseline. Multivariable adjusted positive predictors of incident hypoglycaemia over the follow-up were prior anamnestic hypoglycaemia, retinopathy, depression, insulin use and blood glucose self-measurement, but not sulfonylurea use as previously reported for anamnestic or recalled hypogylcaemia. On the contrary, glitazones, DPP-4 inhibitors and GLP-1 analogues were associated with a reduced risk of hypoglycaemia.

**Conclusions:**

Hypoglycaemia is a frequent adverse effect in ambulatory patients when antidiabetic treatment is intensified. Particular attention is warranted in patients with prior episodes of hypoglycaemia, microvascular disease such as retinopathy and in patients receiving insulin. On the other hand glitazones, DPP-4 inhibitors and GLP-1 analogues are associated with a reduced risk.

## Background

Hypoglycaemia is a serious adverse effect while using antidiabetic pharmacotherapy, irrespective of its severity. Even in cases of less severe hypoglycaemia a substantial reduction of cognitive and motor function as well as hormonal counter regulation is observed [1]. With many antidiabetic drugs such as sulfonylureas or insulin, intensified blood glucose lowering has been associated with an increase in the rate of hypoglycaemia [2-4]. It has been suggested that severe hypoglycaemia may simply be a marker of an increased risk of death and other adverse clinical outcomes rather than a direct cause of such outcomes [5]. The presence of coexisting conditions could increase a patient’s vulnerability to both severe hypoglycaemia and an adverse clinical outcome in the absence of a direct causal link between the two [6,7].

To assess the incidence of hypoglycaemia in a cohort of type-2 diabetic patients in whom treatment was intensified because of insufficient glycaemic control on one or two oral antidiabetic drugs the prospective registry DiaRegis was conducted. The primary objective was to determine the proportion of patients with at least 1 episode of severe hypoglycaemia (requiring medical help or hospitalization) within one year. Hypoglycaemia related secondary objectives were to evaluate the number of patients with at least 1 episode of severe, moderate or mild hypoglycaemia, and to evaluate the number of hypoglycaemic events per patient, respectively.

## Methods

DiaRegis is a prospective, observational, multicenter registry that included 3810 patients with type-2 diabetes under the patronage of the foundation “Der herzkranke Diabetiker” and with sponsorship by AstraZeneca and Bristol-Myers Squibb [8-12]. It is conducted in accordance with Good Epidemiology Practice (GEP), and applicable regulatory requirements. The protocol of this registry was approved by the ethics committee of the Landesärztekammer Thüringen in Jena, Germany on March 4^th^ 2009. Patients being enrolled into this registry provided written informed consent.

### Principal design

The principal design characteristic of DiaRegis was that only patients on one or two oral antidiabetic drugs were enrolled, in which the treating physician intensified treatment at the baseline visit. This was done by either increasing the dose of originally prescribed drugs and / or by exchanging drugs and / or by adding further drugs to the previously used ones. These patients were followed for 12 months to observe which patients developed episodes of hypoglycaemia and to determine patient, disease or treatment characteristics that predicted the development of hypoglycaemia.

### Physicians

Physicians (general practitioners, internists, practitioners and diabetologists) were selected based on a conditioned random sampling method. A physician database with about 9.350 office based physicians treating patients with type 2 diabetes were approached in writing, and physicians with at least 150 patients with type 2 diabetes under regular medical care and with a random distribution across all German regions were asked to participate.

### Patients

Patients with type-2 diabetes at an age of at least 40 years on one or two oral antidiabetic drugs (no injectables such as insulin or GLP-1 analogues) were eligible for inclusion in which the treating physician indicated a change of therapy to be necessary. The inclusion was based on the treating physician’s decision but physicians were asked to consecutively enrol eligible patients. Those not under regular supervision of the treating physician for the duration of the study, those with type-1 diabetes, pregnancy, diabetes secondary to malnutrition, infection or surgery, with maturity onset diabetes of the young, known cancer or limited life expectancy, acute emergencies, participation in a clinical trial and patients with further reasons that made it impossible or highly problematic for the patient to participate and come to the follow-up visits were excluded from participation.

### Documentation

Patient data at baseline were entered via a secure website directly into an electronic database at the *Stiftung für Herzinfarktforschung*, Ludwigshafen, Germany. At this stage, they were automatically checked for plausibility and completeness. For an overview on data collected see the design and baseline publication [8]. Data from the patient questionnaire (paper version) which was asked to be completed by the patient during the visit were transferred to the CRO appointed. The questionnaires were scanned and transferred to the Institut für Herzinfarktforschung for evaluation. All data sets were checked for incorrect data and corrected if applicable; all corrections are documented. All data sets are submitted for biostatistic analysis.

Data on co-morbid disease conditions and risk factors were obtained on an anamnestic basis from the treating physician but diagnoses were not objectively verified independently within this registry. Anamnestic hypoglycaemic events were collected for the last 12 months prior to inclusion based on patient recall. Incident hypoglycaemia was collected by a patient diary which patients were supposed to show at each physician visit.

Blood glucose monitoring was collected as either “yes” or “no”. Given that patients indicated to measure BG themselves, they were asked whether this was done on a daily, weekly or monthly basis and how often.

### Hypoglycaemia definitions

Crude hypoglycaemia rates are reported for anamnestic (any recalled hypoglycaemia within the last 12 months) and incident hypoglycaemia (new episodes of hypoglycaemia within 12 months of follow-up). In addition, hypoglycaemia was classified as follows: in case of severe hypoglycaemia patients were seeking medical attention or were admitted to hospital because of hypoglycaemia. In case of moderate hypoglycaemia patients experienced symptoms of hypoglycaemia and required assistance from another person (e.g. a relative or friend), but no attention of a medical professional was necessary. Mild hypoglycaemia was determined from blood glucose measurements (<2.22 mmol/l; 40 mg/dl in any case; 2.22-2.78 or 50 mg/dl in case of symptoms) and defined as being with or without specific symptoms but manageable without external help [13,14].

### Statistical analysis

For the present analysis on the incidence of hypoglycaemia a total of 3347 out of 3810 initially enrolled patients were available. 463 patients were excluded from the analyses because they were either not alive, had no data available on hypoglycaemia incidence or were lost to follow-up.

The statistical analysis was performed using SAS, version 9.2 (Cary, North Carolina, U.S.A.). The distribution of metric variables is described with medians and quartiles or means with standard deviations. All descriptive statistics are based on available cases. Differences in patient characteristics, risk factors at baseline and concomitant pharmacotherapy were tested using the χ^2^ or Mann–Whitney-Wilcoxon Test.

Stepwise multivariable logistic regression analysis was used to determine independent predictors (adjusted odds ratios [OR] with 95% confidence intervals) for incident hypoglycaemia. Variables entered into the model were identified from univariate analysis, selected for their potential impact on hypoglycaemia and included anamnestic hypoglycaemia, age, diabetes duration, HbA1c, anamnestic heart failure, anamnestic non-proliferative retinopathy, anamnestic proliferative retinopathy, anamnestic coronary artery disease, blood glucose self-measurement, and anamnestic evidence of clinically relevant depression. On the other hand peripheral arterial disease, amputation, heart failure, and peripheral neuropathy were not considered for the multivariable adjustment. In addition a number of antidiabetic drugs such as metformin, sulfonylureas, glucosidase inhibitors, glinides, glitazones, DPP-4 inhibitors, GLP-1 analogues and insulin use were considered.

## Results

### Patient characteristics

Patients considered for the present analysis had a median age of 66.1 (quartiles 51.0-66.1), 46.9% were female, and had median diabetes duration of 5.6 years (2.9-9.4). Median HbA1c was 7.4% (6.8-8.2) and 47.6% had an HbA1c value of at least 7.5%. Patient had a considerable burden of risk factors (dyslipidemia 63.7%, hypertension 84.7%) and co-morbid disease conditions (coronary artery disease 18.2%, peripheral neuropathy 13.6%, heart failure 9.9%, clinically relevant depression 5.3%, and stroke/TIA 4.6%).

### Incidence of hypoglycaemia

**Figure 1:**
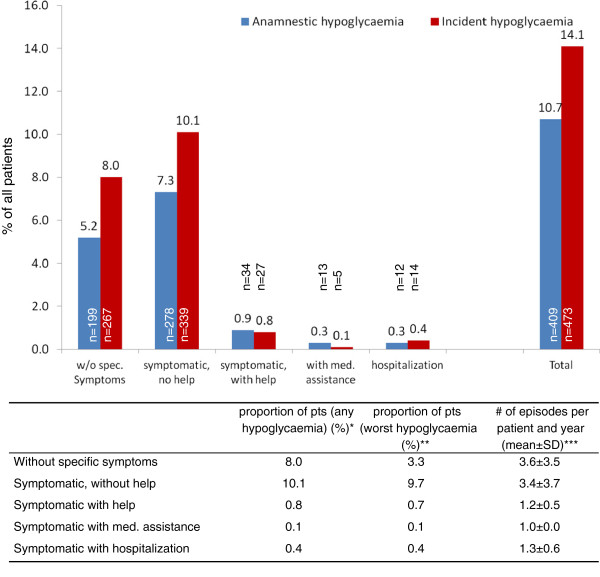
**Anamnestic and incident hypoglycaemia.** Patients with anamnestic hypoglycaemia were confined to those with a 12 months follow-up (n=3347). *Legend.* *multiple episodes with different episodes captured; **only the worst episode of hypoglycaemia per patients included; ***only of those with hypoglycaemia.

Out of a total of 3347 patients 473 (14.1%) had any severity hypoglycaemia over a follow-up of 12 months (Figure 1). This was higher than the rate documented for retrospective hypoglycaemia (11.0%) within the 12 months prior to inclusion (but confined to those with a 12 months follow-up). This higher incidence was mostly was due to higher rates of hypoglycaemia without specific symptoms (8.0 vs. 5.2%) and symptomatic hypoglycaemia but without a need for help (10.1 vs. 7.6%).

The proportion of patients with at least 1 episode of severe hypoglycaemia within one year (primary objective; either requiring medical help or hospitalization) was 0.1+0.4 = 0.5% when only worst episodes were considered (see table in Figure 1, middle column). Furthermore, the proportion of patients with moderate hypoglycaemia (requiring external but no medical help) was 0.7% and 13.0% had mild hypoglycaemia (symptomatic 9.7% or asymptomatic 3.3% but without a need for help) counted as the worst event.

Patients without specific symptoms of hypoglycaemia reported a mean of 3.6±3.5 episodes similar to patients with symptoms but without a need for help (mean of 3.4±3.7 episodes). Severe episodes of hypoglycaemia were reported on average once per year.

An absolute 3.3% of patients had mild hypoglycaemia without symptoms or a need for help (blood glucose values <2.22 mmol/l) (see table, middle column in Figure 1). Overall 10.9% had any degree of symptoms.

### Multivariable predictors of incident hypoglycaemia

Patients with incident hypoglycaemia had longer diabetes duration (6.4 vs. 5.5 years), higher HbA1c values (7.6 vs. 7.4%) at baseline and more frequent anamnestic co-morbid disease conditions such as coronary artery disease, peripheral arterial disease, amputation, heart failure, peripheral neuropathy, diabetic retinopathy (both proliferative and non-proliferative) and clinically relevant depression (Table 1).

**Table 1:** Patient characteristics, risk factors, laboratory values

	**Incident hypo 12 months FU Median (quartiles) or % (n=473)**	**No hypo 12 months FU Median (quartiles) or % (n=2874)**	**p-value / OR (95%CI)**
Age (years)	66.8 (57.8-74.1)	65.9 (57.6-72.7)	0.08
Female gender (%)	43.8	47.5	0.14
Diabetes duration (years)	6.4 (3.0-10.5)	5.5 (2.9-9.2)	<0.01
Blood glucose			
HbA1c (%)	7.6 (6.8-8.8)	7.4 (6.8-8.1)	<0.0001
HbA1c ≥ 7.5%	55.9	46.2	<0.001
FPG (mg/dl)	142 (115–174)	140 (119–168)	0.83
PPG (mg/dl)	183 (156–222)	183 (155–220)	0.39
Blood glucose self measurement (%)	87.7	73.9	<0.0001
Prior smoker (%)	20.9	14.2	<0.001
Bodyweight (kg)	87 (78–98)	89 (78–100)	0.09
Coronary artery disease (%)	22.0	17.5	<0.05
Stroke / TIA (%)	5.7	4.5	0.23
PAD (%)	8.6	5.7	<0.05
Amputation (%)	2.3	0.7	<0.001
Heart failure (%)	13.1	9.3	<0.05
Autonomous neuropathy (%)	4.2	3.4	0.41
Peripheral neuropathy (%)	17.6	12.9	<0.01
NPDR (%)	7.4	3.2	<0.0001
PDR (%)	1.7	0.3	<0.0001
Clinically relevant depression (%)	8.4	4.8	<0.01

*Legend.* Hb_A1c_, glycosylated haemoglobin A1c; FPG, fasting plasma glucose; TIA, transitory ischemic attack; PAD, peripheral arterial disease; PPG, postprandial plasma glucose; (N)PDR, (non) proliferative diabetic retinopathy.

**Figure 2:**
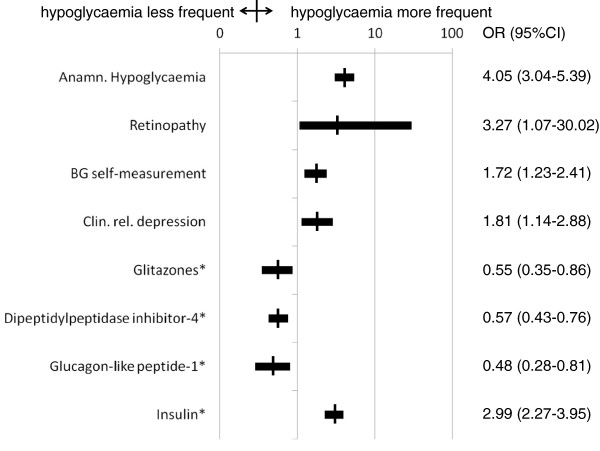
**Multivariable predictors of incident hypoglycaemia. ***Legend.* *in any combination at baseline; BG, blood glucose.

Multivariable adjusted (for variables entered into the model see methods section) positive predictors of incident hypoglycaemia over the 12 months follow-up were prior anamnestic hypoglycaemia (OR 4.05; 95%CI 3.04-5.39), pre-existent retinopathy (3.27; 1.07-30.02), pre-existent clinically relevant depression (1.81; 1.14-2.88), insulin use starting at baseline (2.99; 2.27-3.95) and blood glucose self-measurement (1.72; 1.23-2.41) (Figure 2). On the contrary, glitazones (0.55; 0.35-0.86), DPP-4 inhibitors (0.57; 0.43-0.76) and GLP-1 analogues (0.48; 0.28-0.81) were associated with a reduced risk of hypoglycaemia.

### Drug treatment as a predictor of incident hypoglycaemia

Table 2 examines the relation between antidiabetic drug treatment and incident hypoglycaemia more closely. Amongst patients receiving any type of monotherapy, insulin use was the only positive predictor of incident hypoglycaemia (OR 6.59; 95%CI 4.43-9.79) while the reduced rate with DPP-4 inhibitors was statistically only borderline significant (OR 0.52; 95%CI 0.26-1.03). Insulin use was also predicting hypoglycaemia when used in combination with oral antidiabetic drugs (3.65; 2.92-4.58). Oral dual combination therapy (0.44; 0.36-0.54), combination use of metformin with glitazones (0.46; 0.28-0.83), metformin with DPP-4 inhibitors (0.34; 0.25-0.45) and any OAD with GLP-1 analogues (0.47; 0.30-0.74) were associated with a reduced rate of hypoglycaemia.

**Table 2:** Pharmacotherapy

	**Incident hypo 12 months FU Median (quartiles) or % (n=473)**	**No hypo 12 months FU Median (quartiles) or % (n=2874)**	**p-value / OR (95%CI)**
Any OAD monotherapy	14.4	15.6	0.91 (0.69-1.20)
Metformin	9.5	8.7	1.11 (0.80-1.55)
Sulfonylureas	1.3	1.9	0.66 (0.28-1.54)
Glucosidaseinhibitors	0.0	0.2	--
Glinides	1.3	0.7	1.75 (0.70-4.36)
Glitazones	0.4	0.5	0.94 (0.21-4.16)
DPP-4 inhibitors	1.9	3.6	0.52 (0.26-1.03)
**Injectable monotherapy**	**11.0**	**2.8**	**4.32 (3.01-6.22)**
GLP-1 analogues only	0.0	0.9	--
**Insulin only**	**11.0**	**1.8**	**6.59 (4.43-9.79)**
**Dual oral combination therapy**	**33.1**	**52.9**	**0.44 (0.36-0.54)**
Met + SU	13.8	12.5	1.12 (0.84-1.49)
Met + Glucosidaseinhibitors	0.2	1.3	0.17 (0.02-1.22)
Met + Glinides	2.1	2.2	0.95 (0.48-1.86)
**Met + Glitazones**	**2.5**	**5.4**	**0.46 (0.28-0.83)**
**Met + DPP-4 inhibitors**	**12.3**	**29.3**	**0.34 (0.25-0.45)**
SU + Glitazones	0.4	0.3	1.22 (0.27-5.58)
SU + DPP-4 inhibitors	0.8	1.3	0.67 (0.24-1.90)
**OAD + GLP-1 analogues**	**4.4**	**9.0**	**0.47 (0.30-0.74)**
**OAD + Insulin**	**31.8**	**11.3**	**3.65 (2.92-4.58)**
**Other combinations**	**5.3**	**8.4**	**--**

*Legend.* DPP-4, dipeptidylpeptidas inhibitors; GLP-1, glucagon-like-peptide 1; OAD, oral antidiabetic drugs.

## Discussion

The results of this prospective cohort study of patients with type-2 diabetes suggest that there is a substantial risk for hypoglycaemia in patients whose treatment is intensified after failure of oral mono or oral dual combination therapy. This risk is particularly high for episodes of hypoglycaemia that are symptomatic but where patients require no help and these episodes are repeatedly during the year. Multivariable adjusted predictors of hypoglycaemia were prior anamnestic hypoglycaemia (OR 4.05), microvascular disease such as retinopathy (OR 3.27), clinically relevant depression (OR 1.81) and, with respect to pharmacotherapy insulin use (OR 2.99). On the contrary, glitazones (OR 0.55), DPP-4 inhibitors (OR 0.57) and GLP-1 analogues (OR 0.48) were associated with a reduced risk. Although sulfonylureas were clearly linked with anamnestic hypoglycaemic events [9], there was no significant association between SU use and the risk of hypoglycaemia during the 12 months FU.

### Hypoglycaemia rates

Hypoglycaemia is a frequent adverse effect of antidiabetic drug treatment. Its definition is however, even today, still controversial. The practical definition from a clinical viewpoint of severe (requiring external help for recovery) and mild (self-treated) hypoglycaemia, as described in the DCCT [15], has been widely adopted for epidemiological and clinical use. In 2005 the American Diabetes Association (ADA) defined hypoglycaemia as an event accompanied by a measured plasma glucose concentration ≤ 3.9 mmol/l. This plasma glucose threshold was chosen because in non-diabetic people glucose counter-regulation is activated at this level and antecedent glucose concentrations of ≤3.9 mmol/l reduce counter-regulatory responses to subsequent hypoglycaemia [14]. The DCCT definition does however not consider asymptomatic biochemical hypoglycaemia and thresholds for its diagnosis have been suggested to lie somewhere in between 3.5 and 3.9 mmol/l [13], although the exposure to glucose levels of 3.5–4.0 mmol/l is likely to be of little clinical significance. Because of this controversy Swinnen et al. investigated how the level of blood glucose might impact prevalence rates reported [14]. They demonstrated that the ADA definition of ≤ 3.9 mmol/l would result in a high proportion of hypoglycaemic episodes without a particular clinical relevance.

Because of the aforementioned controversy on the correct definition of hypoglycaemia we have chosen to document the broadest range of possible phenotypes of hypoglycaemia in our registry including asymptomatic hypoglycaemia with blood glucose values <2.22 mmol/l or 40 mg/dl, symptomatic hypoglycaemia with a blood glucose <2.78 or 50 mg/dl but without a need for help, symptomatic hypoglycaemia with external lay help, medical help or requiring hospitalization (all irrespective of blood glucose values). This resulted in an incidence rate of 14.1%, the majority because of asymptomatic hypoglycaemia (3.3%) or symptomatic hypoglycaemia but without the need for help (9.7%). Further we had 0.7% of patients with moderate hypoglycaemia and 0.5% were classified as being severe. This corresponds to a rate of 1.2% based on the DCCT definition for severe hypoglycaemia requiring external help for recovery [15]. Reported rates of severe hypoglycaemic events (as per DCCT) in clinical studies vary between 0.4% (standard therapy group in the ADVANCE study [4]) and 3.1% per year (intensive therapy group in the ACCORD trial) [5]. In UKPDS the rates of major hypoglycaemic episodes per year were 0.7% in the conventional group (mean HbA1c 7.9%) vs. 1.4% with glibenclamide and 1.8% with insulin (mean HbA1c 7.0%) [16].

Hypoglycaemia rates were strongly dependent on antidiabetic drug treatment as outlined in Figure 2. While insulin use independently conferred substantial added risk (OR 2.99; 95%CI 2.27-3.95), GLP-1 analogues, glitazones and DPP-4 inhibitors (ORs 0.48, 0.55 and 0.57 respectively) were associated with a reduced risk. This was also subject of a more detailed analysis in Table 2. The risk increase with insulin use is compatible with the results of a number of observational studies such as the UK Hypoglycaemia Study [17], a retrospective questionnaire based study from Denmark [18] and a study by Donnelly et al. [19]. The UK Hypoglycaemia Study [17] found similar incidence rates of hypoglycaemia in those recently started on insulin and those treated with sulfonylureas but reported increased rates (25%) in those with a longer duration of insulin treatment. Rates reported from Denmark were 16.5% in insulin treated type-2 diabetic patients (44 episodes/100 patient years) [18] and 35 episodes/100 patient years of severe hypoglycaemia in the study by Donnelly et al. [19]. On the other hand, insulin sensitisers (glitazones) and incretin based therapies (DPP-4 inhibitors, GLP-1 analogues) have been associated with a low risk of hypoglycaemia. In the ADOPT study rates of severe hypoglycaemia requiring help (0.1%) in patients on rosiglitazone were comparable to those receiving metformin (0.1%) but substantially lower than those receiving the SU glibenclamide. Rates of self-reported symptomatic hypoglycaemia were 10% for metformin and rosiglitazone and 38.6% for glibenclamide [20]. Newer agents based on the incretin system such as DPP-4 inhibitors and GLP-1 analogues have been associated with low risk of hypoglycaemia except when combined with sulfonylureas or insulin [21-23].

Sulfonylureas, which have been associated with a considerable risk of hypoglycaemia were associated with no added risk in the prospective follow-up, contradicting analyses we reported for anamnestic hypoglycaemia prior to treatment escalation at baseline (multivariable adjusted OR of 2.58; 95%CI 2.03-3.29) [9]. The notion of an increased risk with sulfonylureas has previously been reported from the UK Prospective Diabetes Study (UKPDS) [24] where 31% of subjects reporting mild hypoglycaemic symptoms with insulin secretagogues in the first year of use and from the ADOPT study [20]. A population based study from Germany looking at over 30 000 patients over 4 years found less episodes of severe hypoglycaemia with the newer generation sulfonylurea glimepiride (0.86 vs. 5.6 events with glibenclamide per 1000 patient years) [25]. Against this background our results are somewhat surprising especially in comparison to analyses predicting anamnestic hypoglycaemia in DiaRegis but have to be weighed against a number of changes that were introduced over the course of 12 months: 1) sulfonylurea use was reduced between baseline and the 12 months follow-up from 29.5 to 24.2%, at least partially also because of hypoglycaemia; 2) oral monotherapy went down from 68.4% at baseline to 17.8% at the 1 year FU in favour of combined OAD treatment regimens and insulin / GLP-1 analogue use. This may have masked hypoglycaemia rates seen with sulfonylureas as has been shown to be the case in combination with for example DPP-4 inhibitors [26,27].

Finally rates of hypoglycaemia were lower for anamnestic or recalled episodes than those prospectively collected in the patient’s diary. There are a number of potential explanations for this finding beyond treatment intensification. It appears possible, that is merely reflects a recall bias and retrospective recording of hypoglycaemia may have underestimated the background rate. This is exemplified in individuals with type 1 diabetes who can reliably remember episodes of severe hypoglycaemia after an interval of one year, but recall of mild hypoglycaemia becomes unreliable within a week [28,29]. In people with insulin-treated type 2 diabetes, recall of severe hypoglycaemia is similarly robust over a period of one year [30] but the reliability of recall of mild hypoglycaemia has not been examined and is unlikely to be preserved. In our study, the increase in hypoglycaemia was most marked for episodes which were symptomatic but where no help was required, which are precisely those which may not have been recalled at entry to the Diabetes Registry.

### Predictors of hypoglycaemia

Out of a number of variables considered to be potentially predictive of subsequent hypoglycaemia such as anamnestic hypoglycaemia, age, HbA1c, heart failure, non-proliferative or proliferative retinopathy, coronary artery disease, blood glucose self-measurement, evidence of clinically relevant depression and antidiabetic drug treatment, prior anamnestic hypoglycaemia (OR 4.05), microvascular disease such as retinopathy (OR 3.27) and depression (OR 1.81) were identified to be associated with an increased risk beyond antidiabetic drug treatment as outlined above. On the other hand, the predictive value of a number of variables (except for microvascular disease) identified in a multivariable analysis of the ACCORD dataset [31] was not confirmed which may be related to the selected patient cohort of the ACCORD study with intensified treatment.

A particular role of anamnestic hypoglycaemia has previously been reported from intervention studies such as the Diabetes Control and Complications Trial (DCCT) and a study reporting on the effects of a teaching programme for intensification of insulin therapy where a history of severe hypoglycaemia was one of the main predictors for an increased risk of future severe hypoglycaemia [32,33]. The finding that depression is associated with subsequent episodes of (severe) hypoglycaemia confirms prior data from Finland where depression was also a significant independent risk factor for hypoglycaemia [34]. Furthermore, Williams et al. found in OAD treated type-2 diabetes patients that hypoglycaemia correlated to significantly lower health related quality of life that included more anxiety / depression on a subscale [35].

Blood glucose self-measurement was also predictive for subsequent hypoglycaemic events, most likely because of the increased awareness in cases where episodes were asymptomatic. This sounds like a self-fulfilling prophecy but is important not only because asymptomatic biochemical hypoglycaemia may result in neurological impairment but also because repeated hypoglycaemia blunts symptomatic and hormonal responses to subsequent episodes leading to impaired awareness of hypoglycaemia, also called hypoglycaemia associated autonomic failure (HAAF) [36]. These patients often experience glucose concentrations below 2.0 mmol/l without becoming symptomatic. Furthermore, a number of variables such as glycaemic control, alcohol, exercise, and age affects and reduces symptomatic and hormonal responses to subsequent hypoglycaemia [37-41]. Elderly patients also report different symptoms and responses to hypoglycaemia with less autonomic and more prominent neuroglycopenic symptoms [42]. In this group, hypoglycaemia can be misdiagnosed as dementia or neurological events [43]. We therefore believe that blood glucose self-measurement not only helps to detect “mild” hypoglycaemia but also to detect “asymptomatic but severe hypoglycaemia”.

### Limitations

Among the strength of the dataset the prospective collection of data on hypoglycaemic events after an adjustment of treatment at baseline has to be noted. After the switch patients were followed for one year and data on hypoglycaemia rates, co-morbidity, treatment patterns and outcomes collected over the follow-up supplementing prior, mostly retrospective database analyses. On the other hand there are some limitations to the current analysis deserving consideration: 1) The representativeness for the subset of the German population with type-2 diabetes cannot be assessed. This is because we chose to recruit patients in primary and specialized care but not the general population. This is unlikely to affect the overall conclusion however because it can be assumed that, after an initial period in which type-2 diabetes may be unknown (up to 5 years) most patients in Germany regularly attend physicians for diabetes care. 2) Hypoglycaemia rates, especially those based on laboratory values, are still a matter of debate. This is despite the ADA giving recommendations for its diagnosis based on a threshold of ≤ 3.9%, but others have challenged that the consequence of blood glucose values ≤ 3.9% are clinically irrelevant [13,14]. We have chosen rather low threshold < 2.22 mmol/ especially for those without symptoms in the attempt not to overrate the frequency of these asymptomatic episodes. It may however be challenged based on the aforementioned. 3) Although data of the most important known risk marker of severe hypoglycaemia, impaired hypoglycaemia awareness, was scored in the DiaRegis [8], the completeness of data was insufficient to include this risk marker in the analyses. 4) Finally one might question the selection of variables considered to identify independent predictors of hypoglycaemia. While generally the majority of baseline variables with a significant difference between groups was considered, those with a high likelihood of interference were not selected. On the other hand antidiabetic pharmacotherapy, with only minor differences at baseline were included into the model because of their likely impact on hypoglycaemia rates.

## Conclusions

We conclude that hypoglycaemia is a frequent adverse effect in ambulatory patients when antidiabetic treatment is intensified from oral mono- or dual oral combination therapy. Particular attention is warranted in patients with prior episodes of hypoglycaemia, microvascular disease such as retinopathy and in patients receiving insulin. On the other hand, glitazones, DPP-4 inhibitors and GLP-1 analogues are associated with a reduced risk. Blood glucose self measurement facilitates the detection of very low blood glucose values that are less symptomatic than would be expected.

## Competing interests

Diethelm Tschöpe, Peter Bramlage and Anselm K. Gitt have received research support and honoraria for lectures from a number of pharmaceutical companies including Bristol-Myers Squibb and AstraZeneca, the sponsors of the present registry. Christiane Binz, and Michael Krekler are employees of the sponsor Bristol-Myers Squibb. Evelin Deeg has no potential conflict of interest to disclose.

## Authors’ contributions

DT, PB, CB, MK and AKG have been deeply involved in the conception and design of the study. ED is responsible for the analysis of data. DT and PB have drafted the manuscript and all other authors have been revising the article for important intellectual content. PB serves as the guarantor. All authors have finally approved the version to be published.

## Pre-publication history

The pre-publication history for this paper can be accessed here:

http://www.biomedcentral.com/1472-6823/12/23/prepub
